# Factors Influencing Sex-Related Differences in the Quality of Life of Patients With Atrial Fibrillation: A Systematic Review

**DOI:** 10.7759/cureus.12341

**Published:** 2020-12-28

**Authors:** Komal Moqeem, Mohammad Waseem Beeharry, Tiffany Fang, Khei Jazzle M Lim, Nicholas Tsouklidis

**Affiliations:** 1 Medicine, California Institute of Behavioral Neurosciences & Psychology, Fairfield, USA; 2 Medicine, Royal Surrey County Hospital, Guidford, GBR; 3 Medicine and Surgery, California Institute of Behavioral Neurosciences & Psychology, London, GBR; 4 Trauma and Orthopaedics, Barts Health NHS Trust, London, GBR; 5 Internal Medicine, California Institute of Behavioral Neurosciences & Psychology, Fairfield, USA; 6 Neurosciences and Psychology, California Institute of Behavioral Neurosciences & Psychology, California, USA; 7 Health Care Administration, University of Cincinnati Health, Cincinnati, USA; 8 Medicine, Atlantic University School of Medicine, Gros Islet, LCA

**Keywords:** atrial fibrillation, sex, gender, female, quality of life

## Abstract

Atrial fibrillation (AF) is a growing public health concern, the impact of which is frequently underestimated. It has a profound effect on the quality of life (QoL) which appears to be disproportionately lower in female patients compared to men. We aim to explore the factors contributing to sex-related disparities in the QoL of AF patients by conducting a systematic review using the PubMed electronic search database. We used the following combination of medical subject heading (MeSH) parameters: “atrial fibrillation” and “sex” and “quality of life” with specific inclusion and exclusion criteria. We identified 13 relevant studies published between 2010 and 2020 for our review. These studies evaluated sex-related differences in QoL scores, symptom burden, and AF-related complications originating across different continents in Asia, Europe, and North America. We found that female patients reported a reduced QoL as compared to men and they were more likely to be older with multiple co-morbidities at presentation. Women also reported more frequent and severe symptoms, potentially explained by the greater prevalence of anxiety and depression and thus enhancing symptom perception. Moreover, they were less likely to be managed by anti-arrhythmic medications and invasive rhythm control strategies such as catheter ablation. Female patients with AF experienced more severe strokes, but no sex disparities were found in AF-related cognitive decline. We determined that the more prominent contributory factors towards a lowered QoL in female AF patients appear to be secondary to a higher burden and perception of symptoms as well as under-utilization of invasive treatment modalities. However, further studies are warranted to confirm these findings.

## Introduction and background

Atrial fibrillation (AF) is a growing public health crisis, the magnitude of which is often underappreciated [1]. It is the most common cardiac rhythm disorder characterized by uncoordinated atrial electrical activation leading to ineffective atrial contraction and irregular activation of the ventricles [2,3]. It commonly presents with palpitations; however, it is associated with a range of other symptoms including shortness of breath and fatigue [4]. AF has an estimated prevalence of between 2% and 4%, and this is expected to rise by 2.3-fold due to extended life expectancies and an intensifying search for undiagnosed AF [2,5-7]. It is estimated that there are 5.2 million cases of AF in the United States, and this number is projected to increase to 12.1 million cases by 2030 [6]. In observational studies in Western countries, women have a 30- 50% lower age-adjusted incidence and prevalence of AF, suggesting there some protective factors that contribute to AF developing less readily in women. However, AF incidence increases with age for both sexes. Given the increased longevity of women, the absolute number of men and women with AF is similar on a population basis [8]. Therefore, it is worth identifying any differences in the physiology and potential treatment needs of males and female patients with AF. 

AF has a diverse range of presentations and adverse outcomes, which can contribute to patients' impaired quality of life (QoL). Health-related quality of life (HRQoL) refers to assessing how an individual perceived physical and mental well-being might be affected over time by chronic illness, treatments, and short and long-term disabilities [9]. The concept of QoL has gained significant importance in recent years as an outcome measure of AF and has a central role in the guiding advancement and cost-effectiveness of AF therapies [10]. Several tools are used to assess the QoL in patients with AF, primarily taking the form of questionnaires. These include (1) generic QOL instruments such as the Short Form Survey (SF-12), EuroQoL and Short Form Survey (SF-36); (2) AF specific QoL questionnaires such as Atrial Effect on Quality of Life (AFEQT), quality of life in atrial fibrillation (AF-QoL) and Quality of Life in AF patients (QLAF); and (3) symptom scales for AF such as the European Heart Rhythm Association (EHRA) score [10]. Evaluating QoL forms a relevant component of the comprehensive AF treatment plan. It can help determine the burden of preventable disease, injuries, and disabilities and identify where unmet care needs have to be addressed [9]. Over 60% of AF patients have a significantly impaired QoL, with 17% having disabling symptoms. Although female patients are often underrepresented in AF clinical trials [2,11], available literature suggests that women with AF appear to be more symptomatic and have a lower quality of life [2,3,8,12,13]. This review aims to understand the most significant contributory factors in the sex-rated disparities in QoL measures and why these exist. 

## Review

Methods

Search Strategy

We conducted a systematic review using the PubMed online database and reported findings according to Preferred Reporting Items for Systematic Reviews and Meta-Analyses (PRISMA). The following combination of medical subject heading (MeSH) parameters was entered in the PubMed search engine: “atrial fibrillation” and “sex” and “quality of life.” This yielded 157 articles. 

*Selection Criteria* 

Specific selection criteria were applied to filter the results. Studies were considered for inclusion if they met the following criteria: (1) papers published between January and October 2020; (2) free full text available; (3) papers written in English; 4) studies conducted on human participants. The selection criteria included observational studies (cross-sectional, case report, cohort, etc.), interventional studies (randomized controlled trials, quasi-experimental studies), systematic reviews, and meta-analyses. Exclusion criteria were used to filter out any study which did not use the validated AFEQT questionnaire to evaluate the QoL of AF patients. This was to allow for comparison between studies.

Results

**Figure 1:**
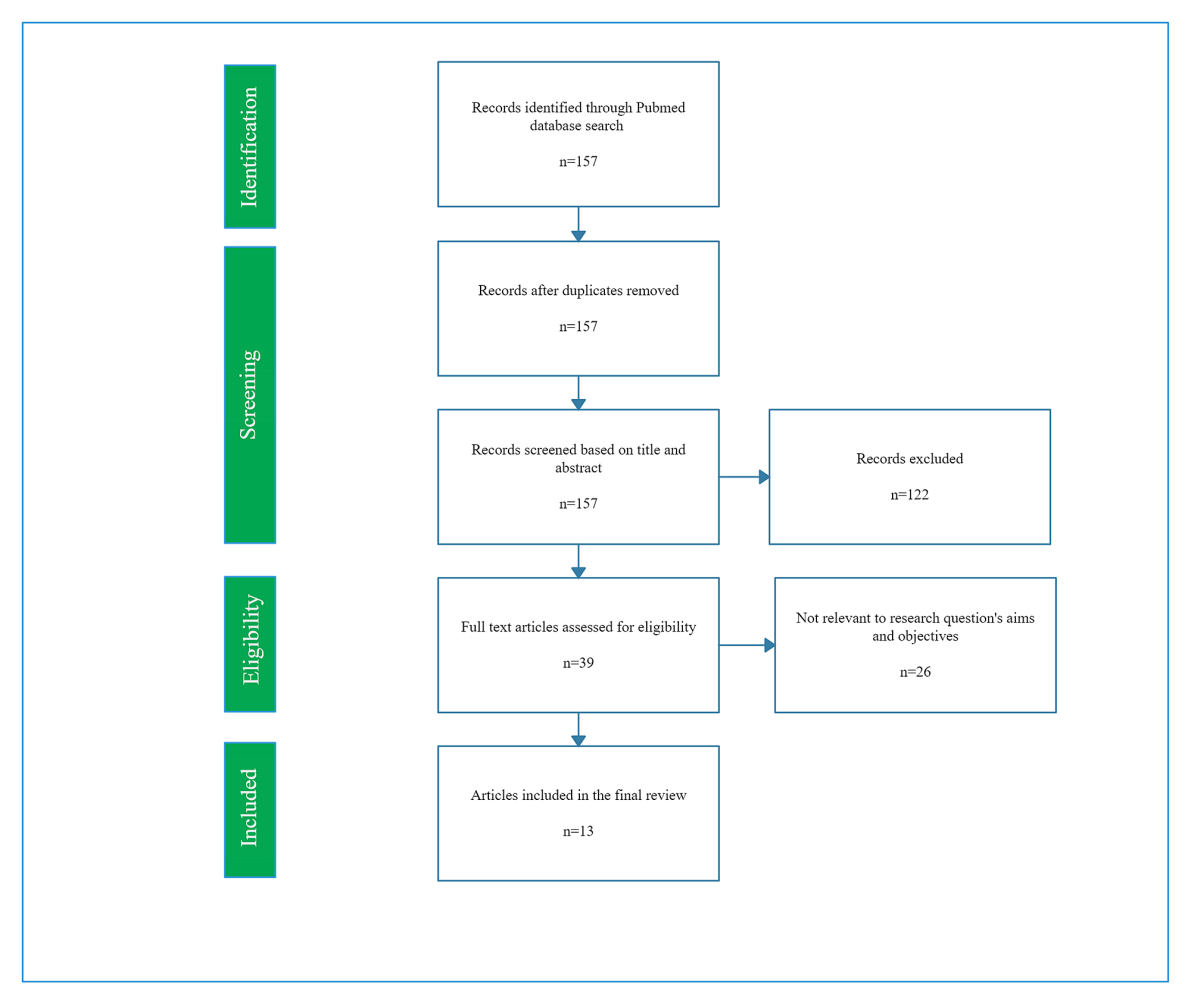
The PRISMA flow diagram of the study selection process PRISMA: Preferred Reporting Items for Systematic Reviews and Meta-Analyses

After the application of all inclusion and exclusion criteria, 39 papers were identified. These 39 papers were systematically evaluated, and 13 studies were deemed suitable for our study specification and met the quality standards required for this review (Figure 1). These included 10 observational studies, two randomized controlled trials (RCTs), and one meta-analysis. The following quality assessment tools were used to assess the standard of the studies: Newcastle Ottawa scale for observational studies; Cochrane Risk of Bias tool for RCTs; and the AMSTAR tool for the meta-analysis. A total of 4,716,788 patients were evaluated across the 13 studies. Seven studies provided data concerning sex disparities in QoL and clinical presentation (Table 1). Three of these seven studies went further and discussed differences in AF therapies in male and female patients. These, along with three additional studies, were used to evaluate sex discrepancies in treatment utilization (Table 2). The last three studies described sex discrepancies in AF related outcomes (Table 3). 

 

**Table 1:** Description of studies concerning sex disparities in clinical presentation and QoL of AF patients AFEQT: Atrial Fibrillation Effect on Quality-of-Life; QoL: quality of life

Sex, Clinical Presentation, and QoL				
Study	Location	Period	Sample	Conclusion
Li et al. (2019) [14]	China	August 2011–December 2016	14,723 patients (5645 38% female)	Women had lower QoL scores as measured by AFEQT score and were more symptomatic compared to men.
Ikemura et al. (2019) [15]	Japan	September 2012–December 2015	1534 patients (458 females 30% female)	Women have lower median AFEQT scores than men in both age groups of <75 years and ≥75 years. The sex gap in QoL as measured by AFEQT scores persisted and grew at the 1year follow-up.
Gleason et al. (2019) [16]	United States	Jan 2011–August 2015	953 patients (35% female)	Female patients were subject to significantly lower QoL as measured by AFEQT scores.
Randolph et al. (2016) [17]	United States	June 2010–August 2011	2007 (43% female)	Women had significantly lower QoL based on AFEQT scores. There was no association between age and QoL. However, age was related to a significant step-wise decline in functional status.
Schnabel et al. (2017) [18]	Western Europe	January 2012–January 2014	6412 patients (39.7% women)	Female study participants reported more severe symptoms and a higher frequency of symptoms compared to men as evaluated by EHRA scores.
Ball et al. (2013) [19]	Australia	2012	2438 patients (48.1% female)	Women have a higher burden of comorbidities at presentation and were more than twice as likely to live alone, and were nearly six times more likely to be widows.
Kupper et al. (2013) [20]	Netherlands	October 2007–March 2009	118 patients (40% women)	AF patients with higher depression levels reported significantly more AF symptoms and reported symptoms to occur at a higher frequency.

**Table 2:** Description of studies concerning sex disparities in AF treatment utilization AF: atrial fibrillation; QoL: quality of life; MOCA: Montreal Cognitive Assessment

Sex and Treatment Utilisation				
Study	Location	Period	Sample	Conclusion
Li et al. (2019) [14]	China	August 2011–December 2016	14,723 patients (5645 38% female)	In the Chinese population, women tended to receive less AF ablation and were less likely to be treated with rhythm control strategies.
Ikemura et al. (2019) [15]	Japan	September 2012–December 2015	1534 patients (458 females 30% female)	Women were less likely to undergo catheter ablation for AF and were less likely to be treated with a rhythm control strategy within the first year of presentation.
Schnabel et al. (2017) [18]	Western Europe	January 2012–January 2014	6412 patients (39.7% women)	Women were more symptomatic but less likely to receive invasive rhythm control therapy, such as electrical cardioversion or ablation.
Kloosterman et al. (2020) [21]	Europe and North America		633 patients (33% women)	No significant difference in the efficacy and safety of first-time catheter ablation between men and women. Both experienced a similar improvement in QoL and MOCA scores following ablation; however, women were more likely to experience bleeds requiring medical attention.
Deng et al. (2019) [22]	Guangzhou, China	June 2011–August 2016	1410 patients (31.9% females)	Catheter ablation for AF has similar risk of recurrence in male and female patients.
Kuck et al. (2018) [23]	Europe	January 2012–January 2015	750 patients (293 39% females)	After catheter ablation of paroxysmal AF, female sex was associated with an almost 40% increase in the risks of primary efficacy failure and cardiovascular rehospitalization.

**Table 3:** Description of studies concerning sex-related disparities in AF outcomes AF: atrial fibrillation

Sex and AF Outcomes				
Study	Location	Period	Sample	Conclusion
Stroke				
Lang et al. 2017 [24]	Austria	March 2003–January 2016	74,425 patients	Women are more likely to experience more severe AF-related strokes.
Nielson et al. 2018 [25]	Denmark	January 1997–December 2015	239,671 patients (48.7% women)	Female sex is a risk modifier for stroke in patients with atrial fibrillation.
Cardiovascular				
Emdin et al. 2016 [26]		January 1966–March 2015	4,371,714 Patients	Atrial fibrillation is a stronger risk factor for cardiovascular disease and death in women compared with men.

Discussion

This review explores sex disparities in the QoL of AF patients and factors that potentially contribute to the reduced QoL reported by female patients. 

Sex Differences in AF-Related Quality of Life 

The studies selected for this review article used the standardized AFEQT questionnaire and the EHRA scale to evaluate AF related QoL and symptom burden, respectively. The AFEQT questionnaire has been validated to support its use as a QoL outcome measure in patients with AF [27]. This 20-item questionnaire uses a 7-point Likert-type response scale to analyze four domains -- symptoms, daily activities, treatment concern, and treatment satisfaction [27]. An overall AFEQT score is calculated using the first three domains to obtain an overall score ranging from 0 (best health status) to 100 (extreme disability). All studies reviewed have suggested that female patients have a significantly lower QoL as assessed by the AFEQT scores [14-17]. Li et al. studied 14,723 patients recruited from the China AF Registry. They showed that women had lower overall mean AFEQT scores than men in those aged <75 years (59.6±15.0 vs. 64.4±14.2, P<0.0001) and in older groups aged ≥ 75 years (57.5±15.1 vs. 61.2±15.3, P<0.0001) [14]. Ikemura et al. evaluated 1534 patients from the multicentre Keio interhospital Cardiovascular Studies-atrial fibrillation (KiCS-AF) registry, which holds data concerning health status and treatment of patients with newly diagnosed AF patients in Japan [15]. The results showed that women had lower median AFEQT overall summary scores than men in both groups aged <75 years (75 {IQR, 60-85} vs. 80 {IQR, 68-90}, P<0.001) and ≥75 years (74 {IQR, 62-85} vs. 81 {IQR, 69-91}, P<0.001) [15]. Similarly, Gleason et al. (2020) studied 953 participants across four academic centers in the Mid-Atlantic Region in the United States. They revealed that female sex was notably associated with poorer AF-related quality of life as assessed by AFEQT scores (β − 2.89, 95% CI: -5.74, − 0.03) [16]. Randolph et al. (2016) in their study of 2007 patients with AF recruited across 99 sites in the United States, demonstrated that median AFEQT score was 79.6 (62.0-91.7) for women compared with 83.3 (69.4-94.4) for men (P<0.0001) [17]. The lower QoL observed in female patients may be secondary to differences in age, co-morbidities, and symptom burden. 

Women develop AF later than men [8] and this is reflected in the studies we reviewed. In the Framingham Heart study that observed 50-year trends in AF data and evaluated 202,417 person-years, it was reported that 74% of women with AF were over the age of 70 years compared to only 58% of men [8]. However, older age alone does not explain lower QoL in women. Interestingly, studies we reviewed suggested that younger patients were more likely to have an impaired QoL. Randolph et al. reported that patients who were younger than 65 had a lower median AFEQT score and this increased for each subsequent older age group (age <65: 78.7 {58.3-90.7}, ages 65-80: 81.5 {65.7-92.6}, age >80: 85.2 {69.6-95.4}, P<0.0001) [17]. Gleason et al. (2020) found no association between AF-related quality of life and age. However, age-related decline in functional status was significant for individuals under the age of 60 compared to those between 70 and 80 (β − 2.30, 95% CI: -4.11, − 0.50) and older than 80 (β − 6.76, 95% CI: -8.76, − 4.77) reported poorer functional status [16]. This suggests that worse QoL outcomes in female patients despite the older age demographic may be due to general musculoskeletal frailty or a longer time period to acquire a higher burden of co-morbidities. Women were more likely to have higher heart failure rates, hypertension, and renal dysfunction at presentation but lower rates of vascular disease [14,15,19]. The comorbidities present in women may contribute to their worse QoL scores. 

Sex Differences in AF-Related Symptoms 

The lower QoL in female patients may also be explained by their higher symptom burden [14-20]. One of the scores recommended to evaluate AF related symptoms is the EHRA scale [28]. It analyses six key symptom domains consisting of anxiety, dyspnoea, palpitations, dizziness, fatigue, and chest pain. Classification of the EHRA score is as follows: Class I ("no symptoms"), Class II ("mild symptoms," normal daily activity not affected), Class III ("severe symptoms," regular daily activity affected), Class IV ("disabling symptoms," normal daily activity discontinued) [28]. A step-wise, negative association was seen between the different classes of EHRA and the AFEQT score [27]. Recent analysis has also demonstrated that change in one EHRA class translated to a +/- 5 points change in AFEQT score [29]. Results from Li et al.'s (2019) study showed that a higher proportion of Chinese female AF patients experienced ‘severe’ and ‘disabling’ symptoms corresponding to EHRA III and IV classification, respectively as opposed to their male counterparts (35.6% vs. 24.0%, P<0.0001) [14]. Another study by Schnabel et al. (2017) evaluated EHRA scores for 6412 participants across seven Western European countries. They reported that female study participants experienced greater severity and frequency than men [18]. Given that QoL appears to be inversely related to symptom burden, greater symptom severity can lower QoL in women [28,29]. However, the reason why women experience more symptoms compared to men is not clear. 

One possible explanation for the more severe symptoms reported by women is that there may be a sex-related difference in symptom perception. Kupper et al. (2013) illustrated through linear regression analyses that AF patients with higher depression levels reported significantly more AF symptoms (β = 0.44; P< 0.0005) and reported symptoms to occur with a higher frequency (β = 0.51; P<0.0005) [20]. However, both male and female AF patients reported significantly higher levels of anxiety and depression compared to the general population (P<0.001) [20], anxiety and depression seemed to be more prevalent in women [16,19]. From a demographic perspective, women were more than twice as likely to live alone and were nearly six times more likely to be widows [19]. This can potentially result in increased sensitivity to AF-related symptoms in women, translating to higher EHRA scores. Attention to emotional distress, particularly in women, is essential in improving symptom control and QoL measures. 

Sex Differences in AF Treatment Utilization 

The common AF treatment options include either rate control or rhythm control strategies with antiarrhythmic drugs, ablation, or electrical cardioversion [2]. Results from recent large-scale randomized control trials have shown that in patients with symptomatic AF, catheter ablation was superior to medical therapies in leading to clinically significant improvements in QoL outcomes [30,31]. However, there appears to be a discrepancy in the utilization of treatment modalities between the two sexes. Li et al. found the use of electrical cardioversion was similar in both sexes within the Chinese population, but women were inclined to receive AF ablation less frequently (5.7% vs. 6.7%, P=0.025) in those aged <75 years [14]. Similarly, Ikemura et al. showed that women were less likely to undergo catheter ablation for AF within the year after presentation in contrast to men (adjusted HR, 0.77 {95%CI, 0.62-0.95}, P=0.02) and were less likely to be treated with a rhythm control strategy (48.1% {n = 214} vs. 58.0% {n = 621}, P<0.001) [15]. They also found that at one-year follow-up, the sex gap in AFEQT scores became wider, suggesting that men were more likely to benefit from AF therapies [15]. The average change in overall mean AFEQT score within one year was 4.97 (95% CI, 3.74-6.20) in women and 10.20 (95% CI, 9.50-11.05) in men [15]. Schnabel et al.’s study across Western Europe found that at the one-year follow-up, men had more frequently received electrical cardioversion (odds ratio {OR}: 0.78; 95% CI: 0.68-0.90), catheter ablation (OR: 0.72; 95% CI: 0.56-0.94) or surgical ablation therapy (OR: 0.45; 95% CI: 0.22-0.93) in comparison to women [18]. 

It is established that women are more likely to experience life-threatening adverse effects of anti-arrhythmic drugs, impacting their use, and explaining their underuse as a treatment strategy in women [32]. However, it is not evident why catheter ablation techniques appear to be under-utilized in female patients. Both Li et al. and Ikemura et al., in their studies in China and Japan, showed that women were less likely to be covered by health insurance [14,15]. This may potentially result in a delay in women seeking medical help and alternatively accepting a more conservative treatment approach. However, these factors are not relevant in countries where healthcare is free at the point of use, such as the United Kingdom. Women are more likely to be older with multiple comorbidities when diagnosed with AF [14,15,19]. They may have an increased risk profile associated with more invasive procedures such as catheter ablation, which may lead to hesitancy on both the physician for a referral. Discrepancies in patient choice can contribute towards lower rates of catheter ablation in women, and it is worth investigating if women are less likely to accept invasive procedures. Current literature regarding the efficacy of catheter ablation in women compared to men gives mixed results. Kloosterman et al.'s (2020) study of 750 European patients revealed no major difference in the efficacy and safety of first-time catheter ablation and reported that both men and women experience similar QoL and cognitive functioning [21]. However, they highlighted that women experienced more bleeding events requiring medical attention (5.7% vs. 2.1%, P= 0.03) and longer hospital stays compared to men (2.1 ± 2.3 vs. 1.6 ± 1.3 days, P=0.004). A study by Deng and colleagues (2019) comprised of 1410 patients in Guangzhou, China reported catheter ablation for AF has a similar risk of recurrences in men and women [22]. Kuck et al. (2018) showed that following catheter ablation of paroxysmal AF, female sex was associated with an almost 40% increase in the risks of primary efficacy failure and cardiovascular rehospitalization [23]. Overall, there is no convincing evidence that catheter ablation was associated with worse outcomes and the risk of serious complications in women. Therefore, any reversible causes behind the under-utilization of catheter ablation in women need to be explored and addressed as it can improve QoL in female AF patients. 

Sex and AF-Related Complication Outcomes

The AF-related complication outcomes include stroke, heart failure, cognitive impairment or dementia, and mortality. Depression and QoL are also recognized as an outcome measure of AF, and these have already been discussed earlier. A study by Lang et al. (2017) of 74,425 adults with acute ischaemic stroke detected that women are more likely to experience more severe AF-related strokes [24]. Women with AF are also more susceptible to experience strokes [25]. Stroke severity is one of the most important predictors of stroke outcome and QoL of life following it [33]. No sex disparities were noted in the risk of AF-related dementia and cognitive impairment [21]. 

A large scale meta-analyses by Emdin et al. (2016) which evaluated 4 371 714 patients across 30 studies showed that when looked at events per 1000 patient-years, absolute risk increases in AF-related outcomes in women compared with men for all-cause mortality (1.8 {95% CI 1.1-2.6}), cardiovascular mortality (4.3 {95% CI 1.9-7.5}), stroke (3.1 {95% CI 1.1-6.1}), and heart failure (6.1 {95% Cl 2.1-12.7}) [26]. Although a reduced QoL can be a predictor of increased mortality in women, likely, clinical symptoms, and disability rather than an increased risk of mortality risk contributes to lower QoL in women.

Strengths and limitations

One of the biggest strengths of this review is that it includes large-scale studies conducted in Asian populations, which suggests that sex disparities in AF are cross-cultural. This review's limitation was that it consisted of studies published from 2010, and hence potentially useful studies published before this date have been omitted. Only articles available as free text and in English were included, limiting the available pool of studies that can be reviewed. 

## Conclusions

Women experience a lower AF-related QoL. The cause of this may be multifactorial that may include a higher symptoms burden, a greater perception of symptoms, and under-utilization of AF-related treatments. These discrepancies are worth addressed given absolute numbers of men and women affected by AF are similar and the incidence of AF is only going to rise globally as the population ages. Key messages from this review include recognizing the emotional component of AF management alongside the regular therapies and highlighting that age should not deter early referral for catheter ablation in women. 
